# Supplementary material for: VPg Impact on Ryegrass Mottle Virus Serine-like 3C Protease Proteolysis and Structure
Source: Int J Mol Sci. 2023 Mar 10;24(6):5347. doi: 10.3390/ijms24065347 (PMC10048871; doi:10.3390/ijms24065347)
Supplement: Supplementary file 1 [file ijms-24-05347-s001.zip › ijms-2236370-supplementary.pdf]

International Journal of Molecular Sciences

Supplementary Data

VPg impact on ryegrass mottle virus serine-like 3C protease proteolysis and structure

Gints Kalnins[1¶], Rebeka Ludviga[2¶], Ieva Kalnciema[3], Gunta Resevica[3], Vilija Zeltina[3], Janis Bogans[4], Kaspars Tars[1], Andris Zeltins[3], Ina Balke[2*]

[1] Structural biology group, Latvian Biomedical Research and Study Centre, Ratsupites street 1, k-1, Riga LV-1067, Latvia
[2] Plant virus protein research group, Latvian Biomedical Research and Study Centre, Ratsupites street 1, k-1, Riga LV-1067, Latvia
[3] Plant Virology group, Latvian Biomedical Research and Study Centre, Ratsupites street 1, k-1, Riga LV-1067, Latvia
[4] Biotechnology core facility, Latvian Biomedical Research and Study Centre, Ratsupites street 1, k-1, Riga LV-1067, Latvia

* Correspondence: inab@biomed.lu.lv
¶ These authors contributed equally to this work.

```
                   1                                                                                                  100
RGMoV      (1)    ----------------------MYGSIVSMVVQAALAFLLSKDPE-SRTAVRGLLASLTGTALILGWQLLCVLRAWLATSLEKEPEAPEDS-WIDLCGPP
RoMoV      (1)    --------------------MKWLEIILTLISLGGFLWITETTSEPGSNRVLGMRSVVLGYLMLLVWKGLSAWLDSRRVRVESEPQAASES-WLEIQGQP
CyCMV      (1)    -----------------MRTTIMLMLLSISGIGLLEVLSRGAYLGPLVTLACQAALVGLILLELLVLIALFIWRVWLRWNVKLSPVPVKPE-NFASVLNV
CySCV      (1)    -----------------MKIIAMLMLLCISGIGLLEVLSRGAYLRPLVTLACQVALVGMILLELLALIVLFIWRVWLRWNVKLSPVPVKAE-NFASVLNI
 LTSV      (1)    --------------MTLKLWEALKVLVVVSSSSIAASLLLTSQVAAGGLDPMLQSPLALCVISMLVLIVRYAWQNMLLNSVEVRDETPPGM-FINLVGEP
 IYMV      (1)    -----------MASFVAGQLRKGMRMMHLPGHIFLLLG-IWLAGLCSDQLPVTSWWWAIPLTIIVNWIASQAAEDFNRLCAGLKPEPLAKRKFGSMG-NP
 RYMV      (1)    ----------MGSSVVGRFLTRTKETTRVDSHLLIVLAGLIAAAIVSERLPVTASLWAIPSAIIANWIVLSAHESFSRFVEGVEIEPMSTLRYGKVQSAP
 TRoV      (1)    ----------------MLSLRSIVKLIVAALNVMFVVTIGVCARVLAPERPVNWNFVALLLTPVLALIAFELLTELRKWMVYTVKEEDLPAP-LSLDSTP
 BSSV      (1)    VLVFSCRTTRGKRTRALATMSWTTLFVCLSCSNSLMLLLAHLLVSMLSMPAELVAMTSSMFIASLVPSVLFATYYWHSKRVVTWLETATQVRVVRLEGRP
 CfMV      (1)    ------------MGCSVVGNCKSVMLMSRMSWSKLALLVSVAMAAAMTDSPPTLICMGILVSVVLNWIVCAVWQEASELILGVSLEATRPS-PARVIGEP
ArtVA      (1)    ------------------------MKLSNLMTLTMLAGLVSVMIDMYQSPQWSLAYWLSLSLWIAALMLQFAWLSWPRVCLE--PEPVKTELWGEMLGDP
 VTMV      (1)    ------------------------MLSELVQLCLSATMTLALVVSMMLDPSYGWLITMSVMLVSLELTIRPFRRSLDYMKIVVRDEPNEPE-VARIVGAP
 PLYN      (1)    -----------------MTFIDFLLVCLVWNVGALLVTQNTNWLPTWTVPLAVIIQLALICICMLLLSVLYVWRWLTLRSIKVDSANYHQT--YAEVIGC
SCMoV      (1)    --------------MNTDTIRAIFLYWMSLALSMAAVVQLETKVDLS----NHQSLVMTTAILLPALSVLFGLRVWRRWKVVIVKEVNPRE-VINLVGEP
RuCMV      (1)    --------------MHRSP--FGYLLLLISLPAGAVTYDCIRGDCQ-AGPNSLMMALMTLLTTGMWWVVSLSLFWIERQRYRSAEEKISRPKRLRLIGDP
 SoMV      (1)    --------------MHRSP--FGYLLLLISLPAGAVTYDCVRGDCP-AGPNCLMMALMTLFTTGMWWIVSLSLYWIERQRYRSAEEKISRPKRLRLIGDP
SNMoV      (1)    ------------------------MLSQIVHLVLLCQMWAAVIATMILDPKFGWMITSLISLTCLELTLKPIRSLIRYVKVEVGPEPESVE-FGRLVGQP
 SBMV      (1)    --------------MYHPGRSPSFLITLANVICAAILYDIRMGGYQ-PGSLVPIVAWMTPFVTLLWLSASFVTYLYRYARTRLLPEEKVARVYYTAQSAP
 SeMV      (1)    --------------MYHPS-CKGYVLLLANMASFAILVDTFLMGVHNPSHVVPVVALMTLCATPLWLSASYVIYLLRYMLVRLSPEAKQDRVYYTATTAP
SYCMV      (1)    --------------MRHPG-LFTYTLLVVNMASFAILLDSFMMGAYESDHVVPAVALMTLCATVLWLSLSLVSFLYGYVRVQLIPEVKRDRIYYVAQSAP
SCPMV      (1)    --------------MYRPS-CLSYVLLVANMWSFAVCANAFIYGSYDPSHNIPIVALMTLCATGLWLSTSVVSFGIRYVRVRVSPEKTQNRTIYVSSGLP
                                         TMD

                   101                                                                                                200
RGMoV     (77)    RFDPSKGVYGE--VLIDGKQHRVVIQPDFWPLLRSAIN-----IDRDEAAVANSIVSSVAPGKEPGSLVCIQAKDGKVIGMGARVHCGP-ATVLVTAGHV
RoMoV     (80)    QFSPEKGVYAD--VLVDGRTHHVVIQPNFWPYLPTVK------QEREEASVYASPMSSIKPGEEPGSLVCIQDQAGHVVGMGARVHCGS-DSLLLTSFHV
CyCMV     (83)    RLDPTRGIIMD--VLRDDVVIPVIVNPNYWQFLPSPAISR---VDGNEASILGNMMNKVNPGSEPASLVSISNGT-DVVGMGARVNYNG-TTWLLTASHV
CySCV     (83)    RLDPARGIIMD--VLRDDVVIPVIVNPNYWQFLPSPALSR---VDGNEAAILGNMMNKVNPGSEPASLVSISNGT-DVVGMGSRVNYNG-TTWLLTASHV
 LTSV     (86)    KLIPEKGIVMDG-VGEDGKVCKVIVNPQWWRFLPGSAISK---NGEDEAAVLDSVYSSVLTGTEPASLVLLKSGS-QTVGFGARVSYEGCSDYLLTAYHV
 IYMV     (88)    RFDPQVGYVVD----VSHGSDVVTVPLPFSLLSGVAG-SALYRKTVTEAYAGGSMPTPVAPDKLPGCVVTLYHGE-TFLGFGSRVRTPNGQDLLMTNHHI
 RYMV     (91)    RFDPSRGYVVD----VSYNGHVIPVILDFTTTTALSVPQRVNPGVSMEASRGGLPPTSVKLEDVPPSVVVLYHDS-VRLGLGTRVRTPTGRDLLMTNHHI
 TRoV     (84)    RFDPIHGITST--VTVDGKVYQVVIQPEYWHLVSPNRSQ----DGNKETVCIDRMSTVTPAGKEPPSLVTLKVGD-RVVGMGSRVSWGG-NTYLLTAAHV
 BSSV    (101)    MLDMSLGVCIPG--IDEHGSCSVVINPEYWNLLPAELS----EAYKNETVVIGCHASKVPPSNLPRSLVWLKTQS-NVVGLGCRVSWGK-ETYLLTAYHV
 CfMV     (88)    VFDPRYGYVAP--AIYDGKSFDVILPISALSSASTRK-------ETVEMAVENSRLQPLESSQTPKSLVALYSQD-LLSGWGSRIKGPDGQEYLLTALHV
ArtVA     (75)    EFDPAKGIVAS--VLYGTETIKVVIQPKWWQYFSSDVLR-----SEKEGACIGSPVSLIEPGKEPNYLVVIQDENGFTRGMASRVQIGR-NDVMLTAFHV
 VTMV     (76)    TYHPIHGVVCK--CLWNSKEFNVVVQPSLWPLAFPPNISQG---VVKEGAMIGVPFTSLPSGAEPKSLVVLYNDG-VRIGMGSRVNWQG-ADYLLTASHV
 PLYN     (82)    RLVPEKGLVMD--ILDGEKLTPVLVNPNYWHLLPDCAISN---KYGTECPVMGVSLSHVAPGKEPSSLVAISNGS-EIIGFGSRVKFNG-VTYLLTANHV
SCMoV     (82)    FLDPVRGVLMNG-ISTSGGTFEVLIEPKWWHLFPRSAISK---DGEKECAIFNAGYSSVLPGTEPTSLVMLKAKD-LAVGFGARVRFNGCSDYLLTAYHV
RuCMV     (84)    YLDPCDGIVGKILDDCTGLVQEVVIQPKWWRFISMSSPTIN-QDEDNECAILGNSYSQVVPRSEPGSFVLLKVNE-EVVGAGCRVIYDG-GDYLLTAHHV
 SoMV     (84)    YLDPCDGIVGKILDDCTGSVQEVVIQPKWWRFINMSSPTVN-QDEDNECAILGNSYPKVEPHREPGSFVLLKVNE-EVVGAGCRVIYDG-GDYLLTAHHV
SNMoV     (76)    TLDPVRGITSK--CIYKNMEFQLVIQPNWWPLVYPAAVSQAS--GIKESAVIGSSFSVVRENAEPASLVTIYNGD-SRVGMGSRVSYNK-ENYLLTAHHV
 SBMV     (86)    YFDPALGVMMQFAPSHGGASIEVQVNPSWISLLGGSLKING-DDASNESAVLGSFYSSVKPGDEPASLVAIKSGP-QTIGFGCRTKIDG-DDCLFTDNHV
 SeMV     (86)    YFDPALGVMMQFAPSSGGPSIEVQINPSWISLLGSSLKLNV-GDASNESAVLGSFYSPVKAGDEPASLVAIKSGP-TTIGFGCRTKIDG-EDCLLTAHHV
SYCMV     (86)    YFDPTLGVMMKFAPSHGGPSIEAQVNPSWISLLDRSLKING-DEQSNESAILGSFYSAVKPGDEPASLVAIKSGA-HTIGFGCRTKIDG-GDVLLTANHV
SCPMV     (86)    HFDPVYGVVKKCEPMGGGPAIELQVNPSWIHLLPTSPAINK-VEVGQESAILGSTYSVVETGGEPKSLVAVKSGD-STLGFGARVYHEG-MDVLMVPHHV
                                         TMD
```

Figure S1. Sobemovirus Pro N-terminal part aa alignment. Artemisia virus A (ArtVA_NC_017914), blueberry shoestring virus (BSSV_NC_029578), cocksfoot mottle virus (CfMV_NC_002618), Cymbidium chlorotic mosaic virus (CyCMV_NC_027123), Cymbidium chlorotic spot virus (CyCSV_KR996515), Imperata yellow mottle virus (IYMV_NC_011536), lucerne transient streak virus (LTSV_NC_001696), papaya lethal yellowing virus (PLYV_NC_018449), ryegrass mottle virus (RGMoV_EF091714), Rottboellia yellow mottle virus (RoMoV_NC_027198), Rubus chlorotic mottle virus (RuCMV_NC_011187), rice yellow mottle virus (RYMV_NC_001575), southern bean mosaic virus (SBMV_NC_004060), subterranean clover mottle virus (SCMoV_NC_004346), southern cowpea mosaic virus (SCPMV_NC_001625), Sesbania mosaic virus (SeMV_NC_002568), Solanum nodiflorum mottle virus (SNMoV_KC577470), sowbane mosaic virus (SoMV_GQ845002), soybean yellow common mosaic virus (SYCMV_KX096577), turnip rosette virus (TRoV_AY177608), velvet tobacco mottle virus (VTMoV_NC_014509). **RAW** & **EAK**: start position of *E. coli* expressed Pro; underlined: transmembrane domain (TMD); **EA**: protease cleavage site for TMD.

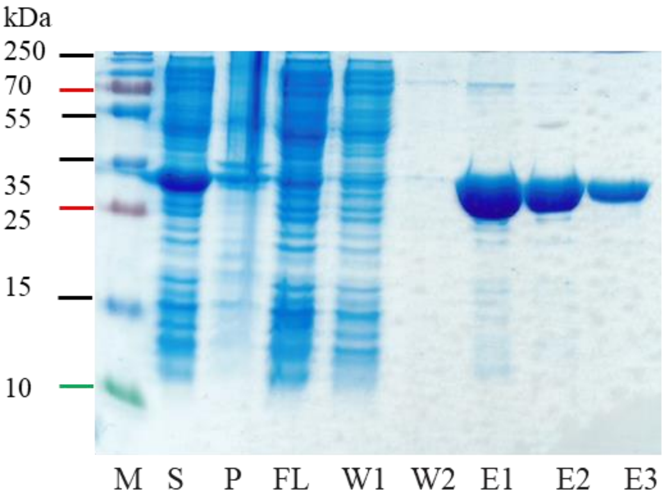

Figure S2. Δ50Pro^{cm} cleavage test *in cis*. M: protein marker (Page Ruler Plus, Thermo Fisher Scientific); S: soluble protein fraction; P: pellet; FL: flow fraction after IMAC column; W1: first wash; W2: second wash; E1: first elution fraction; E2: second elution fraction; E3: third elution fraction.

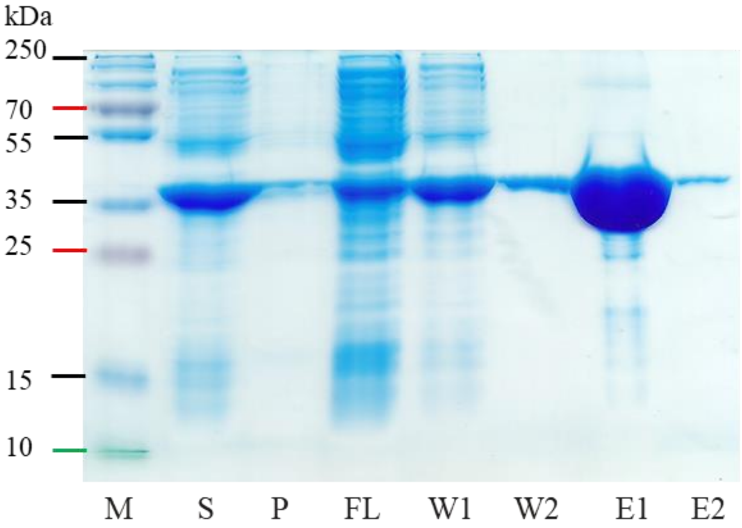

Figure S3. Δ50Pro^{cm}-VPg cleavage test *in tans*. M: protein marker (Page Ruler Plus, Thermo Fisher Scientific); S: soluble protein fraction; P: pellet; FL: flow fraction after IMAC column; W1: first wash; W2: second wash; E1: first elution fraction; E2: second elution fraction.

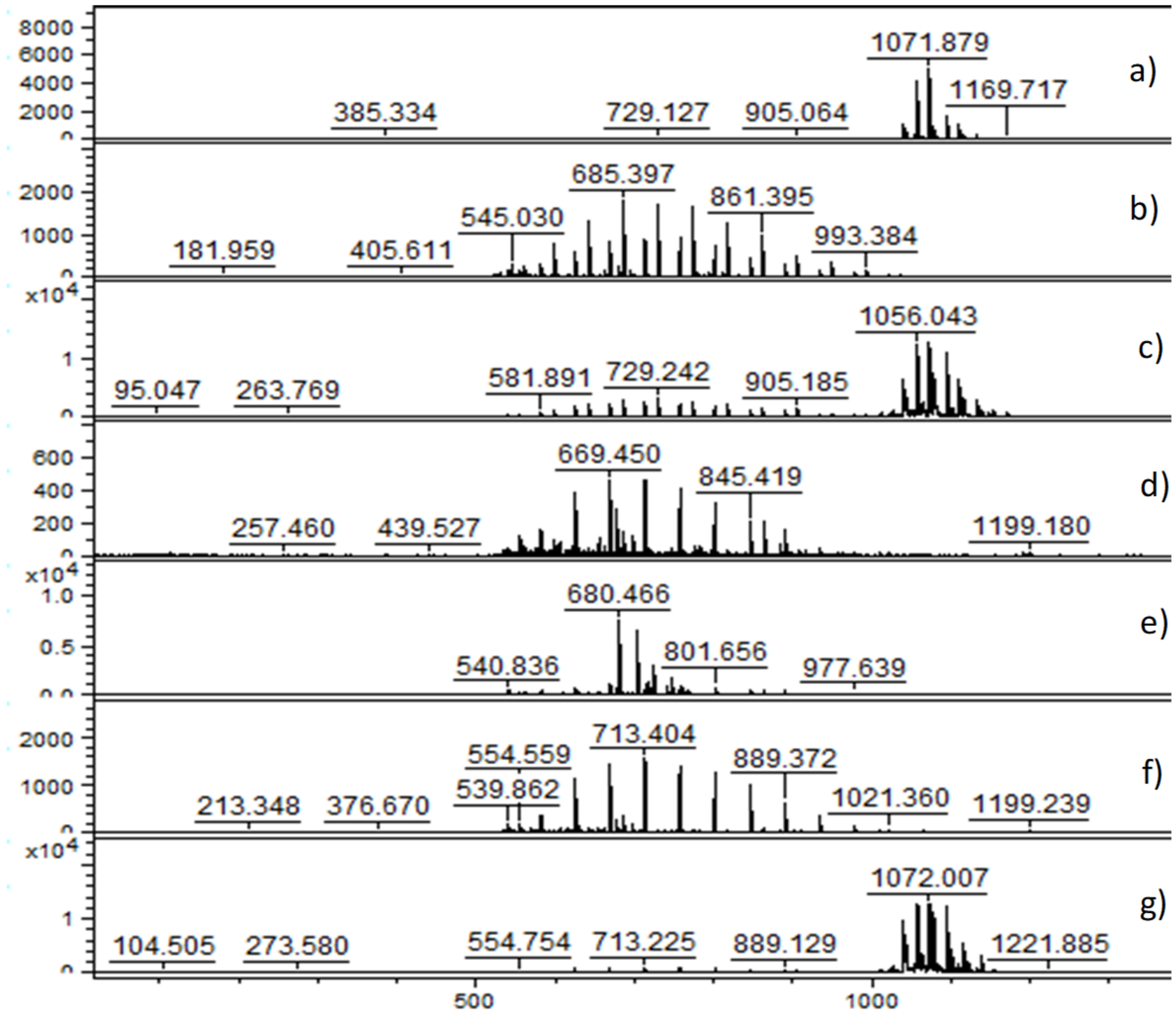

Figure S4. Mass spectrometry analysis of peptide cleavage. A: patterns of the synthetic peptide (Abz-RDE/AAVK(Dnp)-NH_2, MW 1072.9 Da, Pepscan) in the peptide range; c: patterns of Δ117Pro^{cm} (S^{159} was mutated to A) in the peptide range; d: patterns of the synthetic peptide and Δ117Pro^{cm} in the peptide range; e: patterns of Δ117Pro in peptide range; f: patterns of the synthetic peptide and Δ117Pro in the peptide range; g: patterns of the synthetic peptide and Δ117Pro in the peptide range with 20 mM DTT.

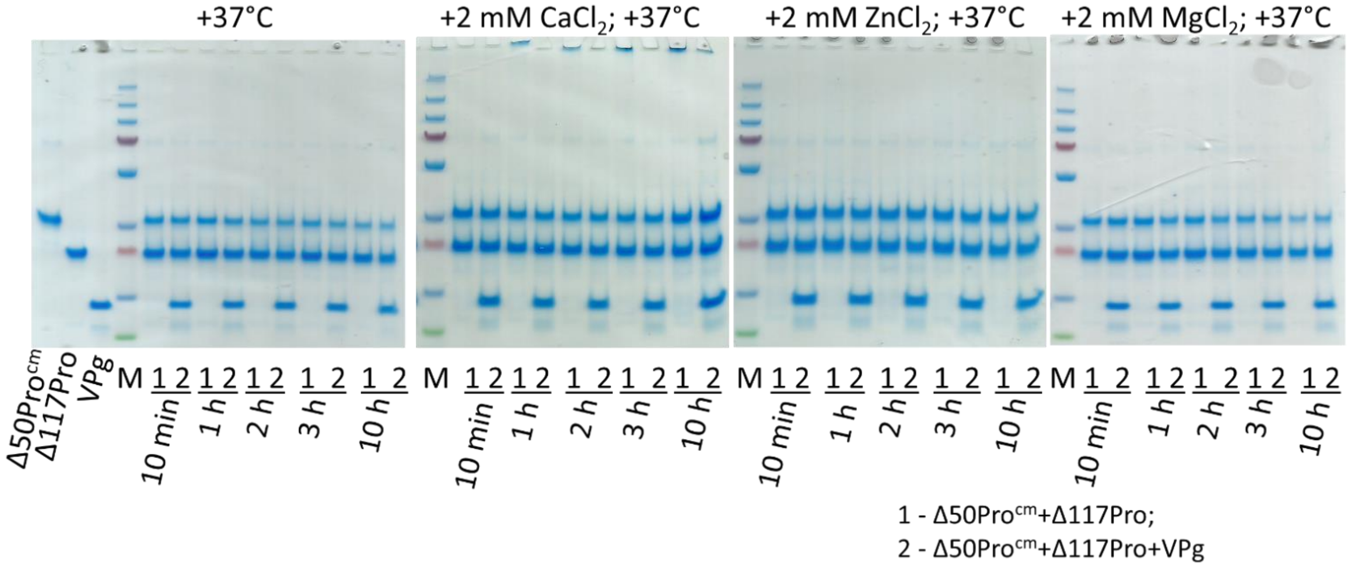

Figure S5. *In vitro in cis* cleavage test of Δ50Pro^{cm}, Δ117Pro and VPg. M: protein marker (Page Ruler Plus, Thermo Fisher Scientific).

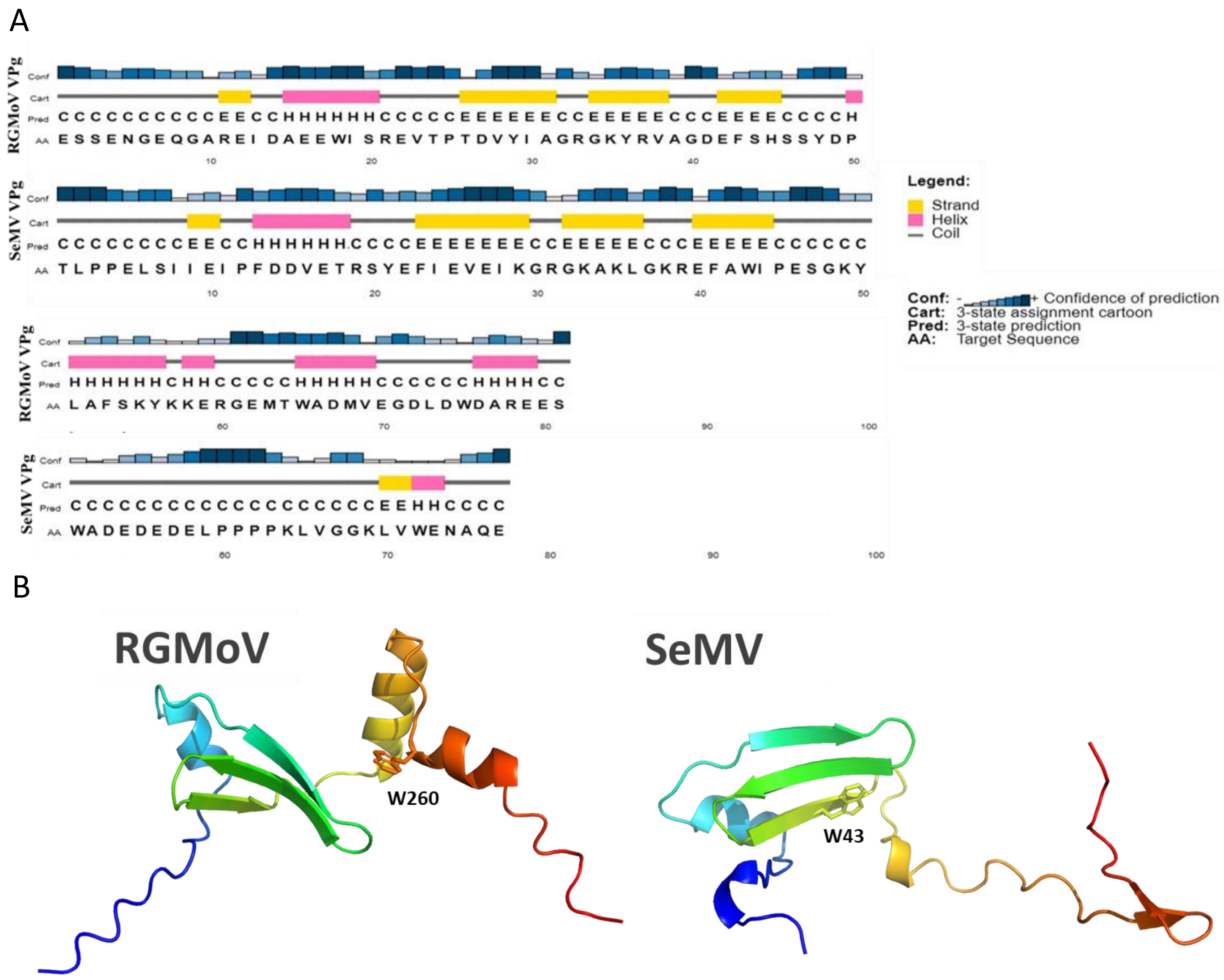

Figure S6. ***In silico*** **analysis of RGMoV and SeMV VPg sequences.** A: RGMoV and SeMV VPg secondary structure element determination by PsiPred [1] (**C**: coil; **E**: strand; **H**: helix); B: RGMoV and SeMV VPg 3D structure determination by AlphaFold [2], key interacting residues are selected as a stick model.

References

1. Jones, D.T. Protein secondary structure prediction based on position-specific scoring matrices. *J Mol Biol* **1999**, *292*, 195-202.
2. Jumper, J.; Evans, R.; Pritzel, A.; Green, T.; Figurnov, M.; Ronneberger, O.; Tunyasuvunakool, K.; Bates, R.; Zidek, A.; Potapenko, A., *et al*. Highly accurate protein structure prediction with alphafold. *Nature* **2021**, *596*, 583-589.

Table S1. List of used oligonucleotides (primers).

Construct	Primer	Primer sequence
N6H-Δ50Pro-E/A-VPg	RG-SerP-RAW-NdeI-F	5′ CCA TAT GCG CGC TTG GCT AGC AAC CTC TCT GGA 3′
	RG-SP-VPg-E/A-R	5′ TCC CTG CTC TCC GTT TGC ACT GGA TGC ACA GTT TGC ATG GAA AAT 3′
	RG-SP-VPg-E/A-F	5′ ATT TTC CAT GCA AAC TGT GCA TCC AGT GCA AAC GGA GAG CAG GGA 3′
	RG2-VPg-HindIII-R	5′ AAG CTT TCA TTC TTC ACG GGC GTC CCA ATC A 3′
	RG-VPg-P16-EcoRI-F	5′ TGA ATT CTC CCA CTC AAG TTA TGA TCC TCT GGCT 3′
N6H-Δ50Pro-TMD-E/A	RGSP-E/A-R	5′ AGC CAC AGC AGC AGC ATC ACG ATC T 3′
	RGSP-E/A-F	5′ AGA TCG TGA TGC TGC TGC TGT GGC T 3′
	RGSP-XhoI-R	5′ AGT GGA CTC GAG CCC CCA TCC CA 3′
VPg-P16-C6H	RG-P16-C6H-HindIII-R	5′ CAA GCT TTC AGT GAT GAT GAT GAT GAT GAG CTG AGG GGG ACC CCT GGA 3′
	RG2-VPg-NcoI-F	5′ CCA TGG GA AAC GGA GAG CAG GGA GCG CGC GA 3′
VPg-E/A-P16-C6H	RG-VPg-E/A-P16-F	5′ TGA AGC GTC CAC AGG AAA TGA TAT TCC TTT AAA CT 3′
	RG-VPg-E/A-P16-R	5′ AGT TTA AAG GAA TAT CAT TTC CTG TGG ACG CTT CA 3′
	RG2-VPg-NcoI-F	5′ CCA TGG GA AAC GGA GAG CAG GGA GCG CGC GA 3′
	RG-VPg-P16-EcoRI-F	5′ TGA ATT CTC CCA CTC AAG TTA TGA TCC TCT GGC T 3′
Δ50Pro-C6H	Pro-C-6-His-HindIII-R	5′ AAG CTT TCA GTG ATG ATG ATG ATG ATG TTC ACT GGA TTC ACA GTT TGC ATG GA 3′
	RG-SerP-RAW-NcoI-F	5′ CCA TGG CTC GCG CTT GGC TAG CAA CCT CTCTGGA 3′
Δ50Pro-VPg-wt-C6H	VPg-C-6-His-HindIII-R	5′ AAG CTT TCA GTG ATG ATG ATG ATG ATG TTC TTC ACG GGC GTC CCA ATCA 3′
	RG-SerP-RAW-NcoI-F	5′ CCA TGG CTC GCG CTT GGC TAG CAA CCT CTC TGG A 3′
	RG2-SerP-HindIII-R	5′ AAG CTT TCA TTC ACT GGA TTC ACA GTT TGC ATG GAA AAT GGA GA 3′
Δ50Pro-N6H	RG-SerP-RAW-NdeI-F	5′ CCA TAT GCG CGC TTG GCT AGC AAC CTC TCT GGA 3′
	SP-AAV-NcoI-N6H-F	5′ ACC ATG GGC CAT CAT CAT CAT CAT CAC GCT GCT GTG GCT AAT TCC ATC GTCT 3′
Δ117SP^{cm}	SP-S/A-F	5′ ACC CGT CCA GGG TGG GCA GGG ACA CCC CT 3′
	SP-S/A-R	5′ AGG GGT GTC CCT GCC CAC CCT GGA CGG GT 3′
	SP-NCE-HindIII-R	5′ GAA GCT TTC ATT CAC AGT TTG CAT GGA AAA TGG AGA GGT TCGT 3′
Δ23Pro-C6H	RG-SerP-E/S-Nco-F	5′CCA TGG TCC AGG ACT GCG GTG AGA GGG CTG CTA 3′

Table S2. Data collection and refinement statistics.

	**Δ117 Pro** (PDB ID 6FEZ)	**Δ117 Pro-E/A-VPg** (PDB ID 6FFO)	**Δ117 Pro^{cm}** (PDB ID 7YZV)
**Data collection**			
Space group	P 1 21 1	P 21 21 21	C 1 2 1
Wavelength, Å	0.9184	0.96411	0.9184
Unit cell dimensions, Å			
a, b, c (Å)	37.11, 115.99, 57.92	38.94, 67.52, 74.08	112.22 36.95 69.50
α, β, γ (°)	90.00° 105.27° 90.00°	90.00° 90.00° 90.00°	90.00° 126.29° 90.00°
Resolution, Å	2.3 (2.30-2.42)	2.1 (2.1-2.21)	1.6 (1.60-1.68)
R_{merge}, %	12.3 (54.8)	7.7 (38)	14.8 (66)
I/σ	5.3 (2.1)	13.9 (4.2)	6.7 (2.2)
Completeness, %	95.98 (98.69)	99.8 (99.8)	95.9 (97.0)
Redundancy	3.0 (3.1)	5.8 (5.9)	5.7 (5.8)
**Refinement**			
Chains in asymmetric unit	2	1	1
Resolution (Å)	57.99-2.3	49.90-2.10	56.02 – 1.60
$R_{work/free}$ (%)	22.946/29.018	17.623/23.481	22.21/26.27
No. of Rfree set (%)	5.1	5.0	4.75
Average B-factor (Å^2)	45.366	31.683	18.13
R.m.s deviations			
Bond lengths, Å	0.016	0.018	0.0053
Bond angles, (°)	1.976	1.897	0.7979
Ramachandran outliers, %			
Favored	92	95	96
Allowed	6	5	4
Outliers	2	0	0

The numbers in parentheses are for the highest-resolution shell.
